# Granulomatous response to *Coxiella burnetii*, the agent of Q fever: the lessons from gene expression analysis

**DOI:** 10.3389/fcimb.2014.00172

**Published:** 2014-12-15

**Authors:** Delphine Faugaret, Amira Ben Amara, Julie Alingrin, Aurélie Daumas, Amélie Delaby, Catherine Lépolard, Didier Raoult, Julien Textoris, Jean-Louis Mège

**Affiliations:** ^1^Aix Marseille Université, URMITE, UMR CNRS 7278, IRD 198, INSERM 1095Marseille, France; ^2^AltraBio SASLyon, France; ^3^Centre d'Immunologie de Marseille-Luminy, Parc Scientifique et TechnologiqueMarseille, France; ^4^Unité Mixte BioMérieux-HCL, Hôpital Edouard Herriot - Pav PLyon, France

**Keywords:** granuloma, Q fever, *Coxiella burnetii*, BCG, transcriptome, type 1 interferon pathway

## Abstract

The formation of granulomas is associated with the resolution of Q fever, a zoonosis due to *Coxiella burnetii*; however the molecular mechanisms of granuloma formation remain poorly understood. We generated human granulomas with peripheral blood mononuclear cells (PBMCs) and beads coated with *C. burnetii*, using BCG extracts as controls. A microarray analysis showed dramatic changes in gene expression in granuloma cells of which more than 50% were commonly modulated genes in response to *C. burnetii* and BCG. They included M1-related genes and genes related to chemotaxis. The inhibition of the chemokines, CCL2 and CCL5, directly interfered with granuloma formation. *C. burnetii* granulomas also expressed a specific transcriptional profile that was essentially enriched in genes associated with type I interferon response. Our results showed that granuloma formation is associated with a core of transcriptional response based on inflammatory genes. The specific granulomatous response to *C. burnetii* is characterized by the activation of type 1 interferon pathway.

## Introduction

Q fever is a worldwide zoonosis caused by *Coxiella burnetii* (Mege et al., 1997). The primary *C. burnetii* infection leads to isolated fever, pneumonia, or hepatitis in 40% of exposed individuals. *C. burnetii* infection may become chronic in patients with valvular lesions, pregnant women, or immuno-compromised patients. In contrast with acute Q fever where the outcome is usually favorable, chronic Q fever is characterized by a long-term drug treatment and persistent risk of relapses. Interestingly, tissue granulomas are present in patients with acute Q fever. In chronic Q fever, granulomas are absent, replaced by lymphocyte infiltrates (Raoult et al., 2005), suggesting that granulomas play an important role in the resolution of Q fever.

Granulomas, defined as tissue collections of macrophages, are generated in response to various microorganisms (Zumla and James, 1996). Their organization varies according to the type of microorganism. In humans, *C. burnetii*-induced granulomas, which are paucibacillary, are composed of a lipid vacuole surrounded by a fibrinoid ring, the “doughnut granuloma” (Srigley et al., 1985; Travis et al., 1986). In contrast, tuberculous granulomas, which are multibacillary, consist of a necrotic core containing bacilli, enclosed by macrophages and surrounded by lymphocytes (Ulrichs and Kaufmann, 2006).

Granulomas are not static organizations but are characterized by continual remodeling and interactions between cell partners (Ramakrishnan, 2012; Shaler et al., 2013). After initial uptake of microorganisms by resident macrophages, the granuloma formation is initiated by recruiting macrophages and blood-derived myeloid cells. The recruitment of activated T-cells by these nascent granulomas completes granuloma formation, and renders them functional (Egen et al., 2008). The main function of granulomas is to contain infectious agents within a limited area, thus restricting the spread of pathogens. Once the infection is contained, the granuloma cells participate in the destruction of infectious agents. Indeed, wild type mice clear mycobacterial infection through granuloma formation whereas mycobacteria disseminate and granulomas are absent in mice that do not express interferon-γ (Cooper et al., 1993). In the majority of patients with tuberculosis, the presence of calcified granulomatous lesions is associated with a controlled infection (Ulrichs and Kaufmann, 2006). However, in others, mycobacteria induce the necrosis of infected macrophages, resulting in a caseum at the center of granulomas. This accumulation of caseum leads to collapsing granulomas and the spread of bacteria (Russell et al., 2009).

Studying granuloma formation in mice requires invasive methods that are not appropriate for human studies. A method was recently developed to generate human granulomas *in vitro* using peripheral blood mononuclear cells (PBMCs) co-cultured with beads coated with BCG (Puissegur et al., 2004; Delaby et al., 2010) or *C. burnetii* extracts (Delaby et al., 2010). This method enables to follow the initial events of granuloma formation and to investigate the molecular mechanisms of granulomas (Egen et al., 2008; Delaby et al., 2012). In this study, we used a high throughput transcriptomic approach to characterize human granulomas induced *in vitro* by *C. burnetii* and to compare them with those induced by BCG. We found that numerous modulated genes were shared by *C. burnetii*- and BCG-induced granulomas, including chemotaxis-associated genes and M1 genes. *C. burnetii* induced a specific repertoire of upmodulated and downmodulated genes that included the activation of interferon-stimulated genes (ISGs), which confers a new role for this pathway in host response to *C. burnetii*.

## Materials and methods

### Patients with Q fever

The study was approved by the Ethics Committee of the Aix-Marseille University. Written informed consent was obtained from each subject. Four patients with acute Q fever and 5 patients with Q fever endocarditis were selected. The diagnostic of acute Q fever was based on serological determination of anti-phase II *C. burnetii* antibodies (Abs). The suspicion of Q fever endocarditis was based on standardized questionnaire that included pathological evidence of endocarditis, positive echocardiograms, positive blood cultures, high titers of IgG specific for phase I *C. burnetii* (Raoult, 2012). The average age of patients with acute Q fever was 43 years old (ranging from 30 to 57 years old). The average age of patients with Q fever endocarditis was 54 years old (ranging from 40 to 74 years old). Six healthy individuals (with a mean age of 37 years, ranging from 28 to 56 years old) were used as controls.

### Preparation of circulating cells

PBMCs were prepared from leukopacks (Etablissement Français du Sang) or blood collected in ethylene-diamine-tetraacetic acid (EDTA) tubes from donors and patients after centrifugation through a Ficoll density cushion. Monocytes were isolated from PBMCs by CD14 positive selection using magnetic beads coated with anti-CD14 antibodies (Miltenyi Biotec). CD14^+^ monocytes were differentiated into macrophages by cell culture (Ghigo et al., 2010). To obtain M1 macrophages, macrophages were stimulated with 20 ng/mL recombinant human IFN-γ (Tebu-bio) for 18 h. M2 macrophages were obtained by incubating macrophages with 10 ng/mL IL-10 or 20 ng/mL IL-4 (R&D Systems) for 18 h.

### *In vitro* generation of granulomas

Granulomas were induced by using two procedures. First, sepharose beads were coated with bacterial extracts from phase I *C. burnetii* or BCG as previously described (Delaby et al., 2010). PBMCs recovered from leukopacks (2 × 10^6^ per assay) were cultured with 800 coated beads for 8–12 days in the presence of mAbs against CCL2 and CCL5 or control isotypes (R&D Systems). Individual granulomas were then collected by micromanipulation and incubated in 2 mM EDTA, allowing cells to dissociate (Delaby et al., 2012). Second, the granuloma formation in patients with Q fever and healthy donors was determined by incubating PBMCs with *C. burnetii* (Puissegur et al., 2004). PBMCs (2 × 10^6^ cells per assay) were cultured with 2 × 10^7^ heat-killed bacteria (100°C, 1 h) in RPMI 1640 supplemented with fetal calf serum, L-glutamine and antibiotics in 6-well culture plates at 37°C. Cell aggregation was observed every 2 days under light microscopy, and cells were recovered after 8–10 days when the size of aggregates was the highest.

### RNA extraction and microarray

Total RNA was extracted from granuloma cells using the RNeasy Mini kit (Qiagen) and DNAse treatment. The granuloma cell gene expression was analyzed using 45,000 probes microarray chips (4 × 44K whole human genome G4112F, Agilent Technologies) and One-color Microarray Based Gene Expression Analysis kit, as previously described (Ben Amara et al., 2010). Three samples per experimental conditions were included in the analysis. Following array scans, image analysis and correction of intra-array signals were performed with Feature Extraction Software A.10.5.1.1 (Agilent Technologies) using default parameters. Minimum Information About a Microarray Experiment-compliant data are provided in the Gene Expression Omnibus (GEO) (Moal et al., 2013) at the National Center for Biotechnology Information (http://www.ncbi.nlm.nih.gov/geo/), and can be accessed with the GEO series accession number (GSE37666).

### Microarray analysis

Raw signal data were normalized with a False Discovery Rate below 0.1 and an absolute fold change (FC) value of 3.0. All analyses were performed using R software (version 3) with the bioconductor libraries (Gentleman et al., 2004). Functional annotation was performed using ClueGO plug-in (Bindea et al., 2009) and selecting terms belonging to GO Biological (Moal et al., 2013) in Cytoscape software (Smoot et al., 2011). An enrichment/depletion test, along with the Benjamin-Hochberg correction method, was performed for statistic analysis. GO terms from levels 6 to 8 in GO tree were selected, with a kappa score above 0.44, to create functional annotation network. Each node represents a GO term and contains at least 3 genes. The leading group term of a functional group was defined as the group containing the largest number of genes. Identification of biological groups depicted in the pie chart was established by manual selection of articles from Pubmed database, filtered to show those describing gene functions in immune cells. The identification of M1 and M2 signatures in granuloma cells was performed using the gene profiles of macrophages stimulated with IL-4, IL-10, and IFN-γ referenced in Gene Expression Omnibus database (GSE36537).

### Real-time quantitative RT-PCR (qRT-PCR)

Reverse transcription of 100 ng of total RNA was performed as previously described (Ben Amara et al., 2010). Primers were designs using Primer3 (Moal et al., 2013) and their sequences were listed in Table 1. Quantitative PCR was performed with Light Cycler Fast Start DNA master^PLUS^ SYBR Green I (Roche applied Science). The results were normalized with the housekeeping gene β-actin. The FC of target genes relative to β-actin was computed using the formula FC = 2^−ΔΔCt^, where ΔΔCt = [(Ct_Target_ − Ct_Actin_)_stimulated_ − (Ct_Target_ − Ct_Actin_)_unstimulated_)] (Moal et al., 2013). The agreement between qRT-PCR and microarray data was assessed by Pearson correlation coefficient.

### Statistical analysis

Comparisons between two groups were performed using the Mann-Whitney *U*-test.

## Results

### Global gene program of granuloma cells

**Figure 1:**
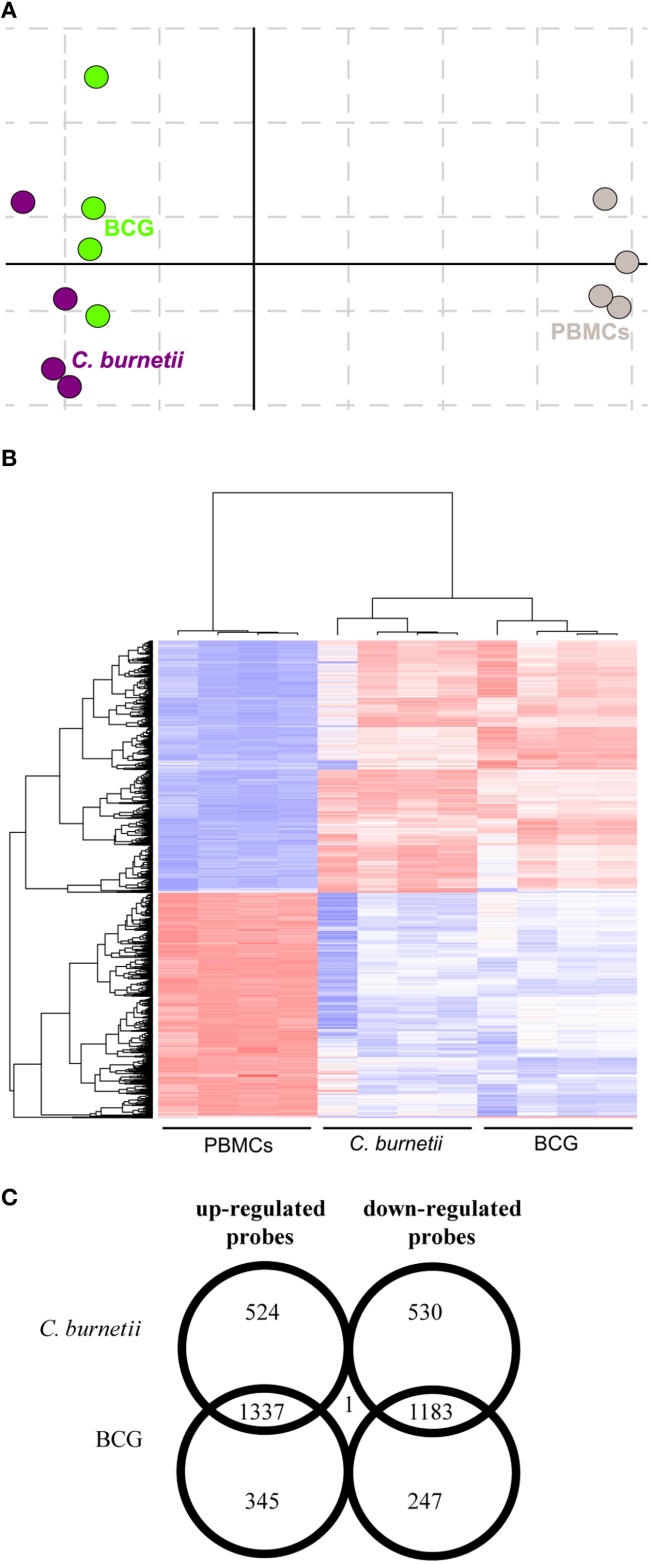
**Transcriptional responses of granuloma cells**. Granuloma cells and PBMCs were analyzed by microarray. **(A)** The correspondence analysis of the probeset signature revealed that *C. burnetii* and BCG-induced granulomas were clearly separated from PBMCs. **(B)** The hierarchical clustering analysis showed specific clusters of upmodulated and downmodulated probes in granuloma cells compared to PBMCs. Gene expression level is color-coded from blue (downregulation) to red (upregulation). **(C)** The Venn diagram represents the expression of upmodulated and downmodulated probes in *C. burnetii* and BCG granuloma compared with PBMCs. The numbers in overlapping regions indicate the number of probes commonly modulated in granuloma cells obtained in response to *C. burnetii* and BCG. The numbers in non-overlapping regions indicate the number of probes specifically modulated in response to *C. burnetii* or BCG.

We previously reported that PBMCs are capable to form granulomas when they are incubated with beads coated with bacterial extracts (Delaby et al., 2012). We wondered if granuloma formation was associated with specific changes in gene expression programs. We compared the transcriptional profiles of granuloma cells with that of naïve PBMCs using whole genome microarrays. Correspondence analysis was conducted to assess the reproducibility of data. The first axis of variance showed large differences between PBMCs and granuloma cells, while the second axis of variance revealed smaller differences between granuloma cells generated in response to *C. burnetii* and BCG (Figure 1A). Hierarchical clustering also showed that the transcriptional responses of granuloma cells were different from that of PBMCs (Figure 1B). Most probes that were upmodulated (1337) and downmodulated (1183) were common in *C. burnetii* and BCG challenges. The probes that were specifically modulated by *C. burnetii* included 524 upmodulated probes and 530 downmodulated probes. Conversely, 345 and 247 probes were upmodulated and downmodulated, respectively, by BCG. The expression of only one probe, the *CD163* gene, increased after *C. burnetii* challenge but decreased after BCG challenge (Figure 1C). These data showed that about 60% of modulated genes were shared by granuloma cells but that the granulomatous responses to *C. burnetii* and BCG were, in part, specific.

### M1/M2 polarization of granuloma cells

**Figure 2:**
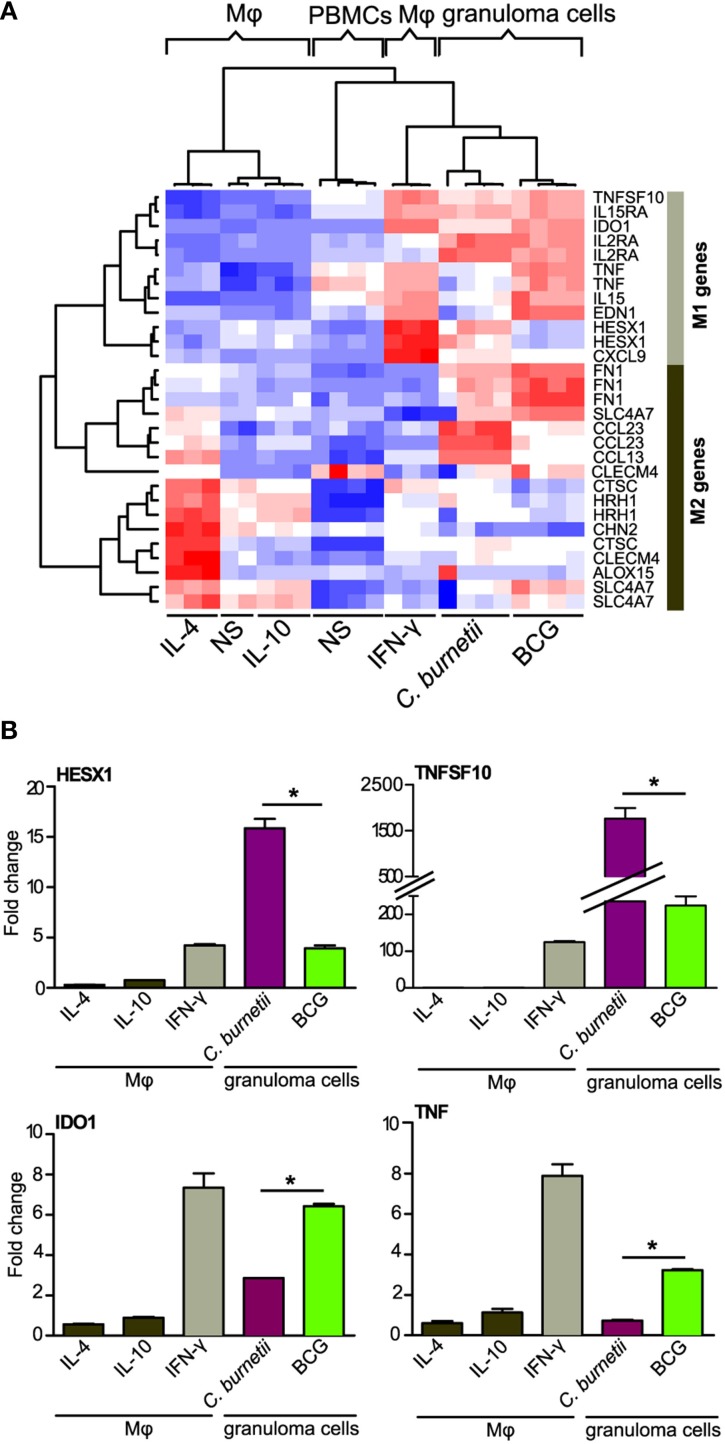
**Atypical M1 polarization of granuloma cells**. The M1/M2 polarization of granuloma cells was studied using macrophages stimulated with IFN-γ (M1), IL-10, IL-4 (M2) as controls of M1 and M2 polarization. Microarrays **(A)** and qRT-PCR **(B)** were performed on M1/M2 macrophages and granuloma cells. **(A)** The hierarchical clustering of specific markers of M1 or M2 polarization indicates that granuloma cells induced by *C. burnetii* and BCG were located within a unique cluster near M1 macrophages. Note that M2 genes were modulated in granuloma cells as well. Gene expression level is color-coded from blue (downregulation) to red (upregulation). **(B)** The expression of *HESX1, TNFSF10*, *IDO1*, and *TNF* (M1 genes) was increased in *C. burnetii*- and BCG-induced granulomas, but their expression was differentially modulated in both types of granulomas. ^*^p < 0.05 for the comparison between *C. burnetii* and BCG using Mann-Whitney *U*-test. M_φ_, Macrophages; NS, unstimulated.

The macrophages are known to be polarized into M1 or M2 cells, which is associated with microbicidal response or permissive response for intracellular bacteria respectively (Benoit et al., 2008). We wondered whether granuloma macrophages, which represent about 40% of granuloma cells (Delaby et al., 2012), were polarized. Nine M1- and 9 M2-related genes (corresponding to 12 and 17 probes, respectively) were selected according to published data (Martinez et al., 2006). Hierarchical clustering analysis showed that granuloma cells clustered with IFN-γ-stimulated macrophages (M1 macrophages) but not with IL-4- or IL-10-stimulated macrophages (M2 macrophages) (Figure 2A), demonstrating that granuloma cells exhibited inflammatory/microbicidal phenotype. This finding was confirmed by the measurement of inflammatory cytokines, TNF and IFN-γ, which were released after 9 days (IFN-γ: 204 ± 53 pg/ml; TNF: 1250 ± 250 pg/ml). However, the granulomatous responses to *C. burnetii* and BCG included some features of M2 macrophages. Indeed, *C. burnetii* upmodulated the expression of *CCL23* and *CCL13* genes, and BCG that of *FN1* and *SLCA47* genes. Other differences were evident: *C. burnetii* caused a slight increase in the expression of *TNF* and *EDN1* genes, but increased greatly the expression of *HESX1* and *CXCL9* genes, in comparison with BCG. To confirm microarray results, qRT-PCR was performed on M1 genes (Figure 2B). The profiles of each gene were significantly correlated in qRT-PCR and microarray (*R*^2^ = 0.64, *p* < 0.008). In addition, the expression of *HESX1* and *TNFSF10* genes was greater in *C. burnetii*-induced granulomas than in BCG-induced granulomas. Conversely, BCG induced a higher increase in the expression of *IDO1* and *TNF* genes than *C. burnetii*. Taken together, these results suggest that granuloma macrophages were rather polarized into M1 macrophages, with subtle differences between *C. burnetii*- and BCG-induced granulomas.

### Functional annotation of *C. burnetii*-specific transcriptional program

**Figure 3:**
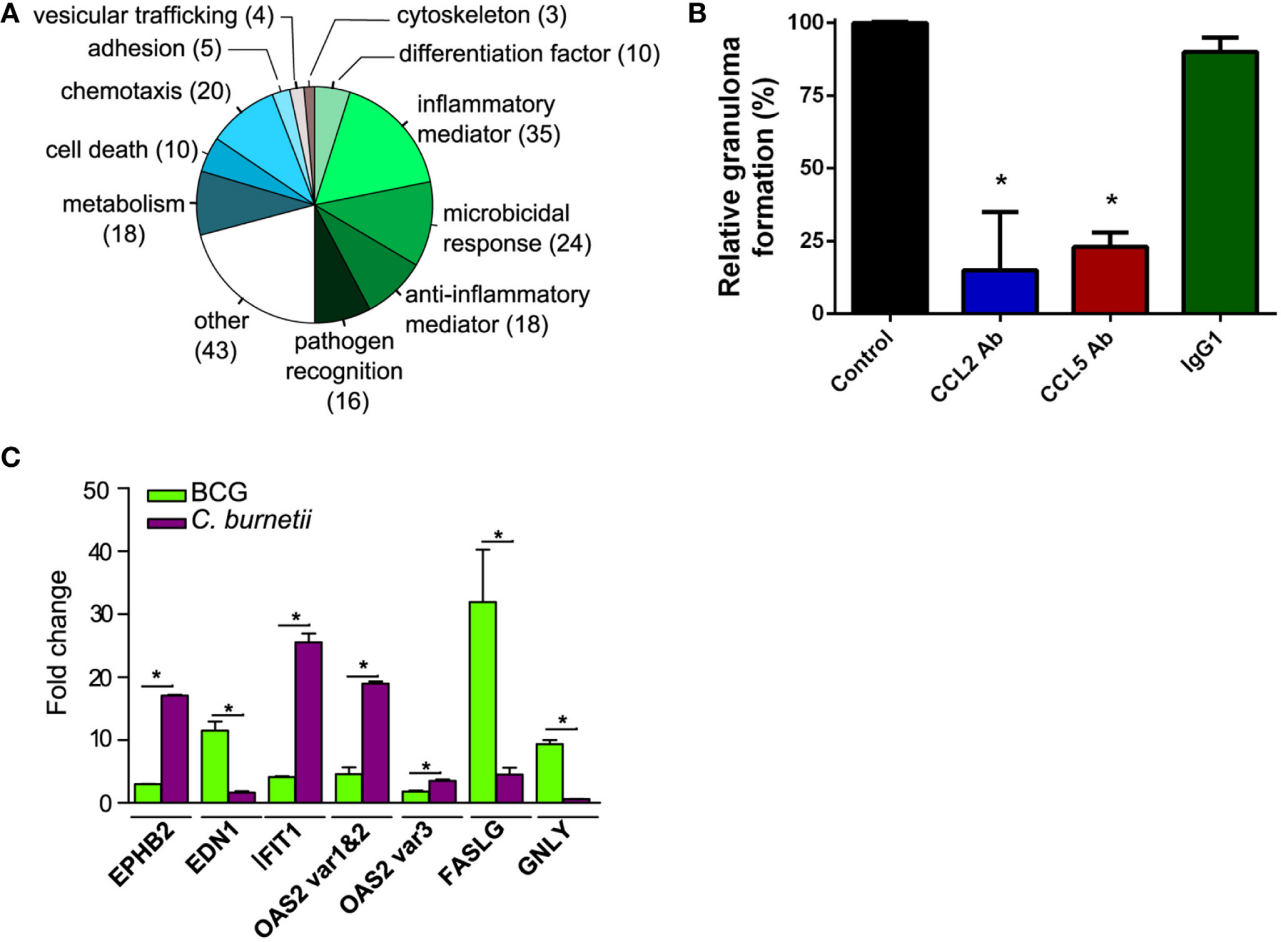
**Genes related to immune response in granuloma cells. (A)** The genes that were modulated in granulomas induced by *C. burnetii* compared with BCG-induced granulomas were manually classified according to functions previously reported in immune response. The pie chart represents the distribution of modulated genes in each biological group. **(B)** PBMCs were incubated with *C. burnetii*-coated beads in the presence of CCL2 Ab, CCL5 Ab, and IgG1 or in their absence (Control) and the formation of granulomas was measured. Results were expressed as relative granuloma formation. ^*^p < 0.05 for the comparison of conditions with and without antibodies. **(C)** The expression of *EPHB2, EDN1, IFIT1*, *OAS2, FASLG*, and *GNLY* genes in granuloma cells was confirmed by qRT-PCR. *p < 0.05 for the comparison between *C. burnetii* and BCG using Mann-Whitney *U*-test.

The gene expression program specifically induced by *C. burnetii* was analyzed by retaining only genes differentially modulated between *C. burnetii* and BCG (absolute *FC* > 3.0). We found that 206 genes were specifically modulated, and their roles were studied using GO Biological Process annotation and according to immune function. Nearly 50% of them were related to inflammatory mediators (17%), microbicidal activity (12%), anti-inflammatory mediators (9%) and pathogen recognition (8%). Other cell functions, such as chemotaxis, cell death, and metabolism were also differentially modulated (Figure 3A). Taken together, the genes that were specifically modulated by *C. burnetii* were organized into functional networks, suggesting a role in granuloma function.

Next, we investigated how these 206 genes were differently modulated in response to BCG or *C. burnetii* relative to PBMCs. A number of genes involved in chemotaxis were similarly modulated in granuloma cells in response to BCG or *C. burnetii* (Figure S1). The genes involved in chemotaxis play a critical role in granuloma formation. This is illustrated by the inhibition of granuloma formation when *C. burnetii*-coated beads were incubated with PBMCs in the presence of mAb directed against CLL2 and CCL5 but not with control isotypes. The mAb directed against CCR5 did not affect granuloma formation (Figure 3B). The functional groups associated with inflammation were differently modulated in granulomas induced by *C. burnetii* or BCG. Many genes related to inflammation were downmodulated by *C. burnetii* and upmodulated by BCG. The genes belonging to anti-inflammatory mediators were weakly upmodulated in response to *C. burnetii* but were strongly downmodulated by BCG. Nevertheless, *C. burnetii*-induced granulomas were not only characterized by anti-inflammatory program; indeed, *C. burnetii* did induce several genes related to inflammation such as *TNFSF13*, *CH25H*, and *IRF7* genes. The expression of genes related to cell death was also decreased in *C. burnetii*-generated granulomas, but increased in BCG-induced granulomas. Finally, *C. burnetii* strongly upmodulated the expression of genes involved in microbicidal response and, especially, ISGs including *MX1*, *MX2*, *IFI44*, *IFI6*, *IFIT1*, *IFITM2*, *IFITM3*, *ISG15*, *OAS1*, *OAS2*, *OAS3*, and *HERC5* genes, whereas BCG had little effect on these genes. The transcriptional differences between granulomas induced by *C. burnetii* and BCG compared with PBMCs were confirmed by qRT-PCR performed on several genes. Indeed, the expression of genes encoding inflammatory mediators such as *EPHB2* gene and *EDN1* gene was highly increased in response to *C. burnetii* and BCG, respectively. The expression of genes involved in cell death, such as *FASLG* and *GNLY*, was increased in response to BCG. In contrast, the expression of ISGs (*IFIT1* and *OAS2*) was highly upmodulated in granulomas induced by *C. burnetii* (Figure 3C). Taken together, these results showed that the granulomatous response shared common features, but also included specific characteristics, according to the nature of the pathogen. They also showed that the granulomas induced by *C. burnetii* were characterized by the activation of type 1 IFN genes.

### Transcriptional response of granulomas in Q fever patients

**Figure 4:**
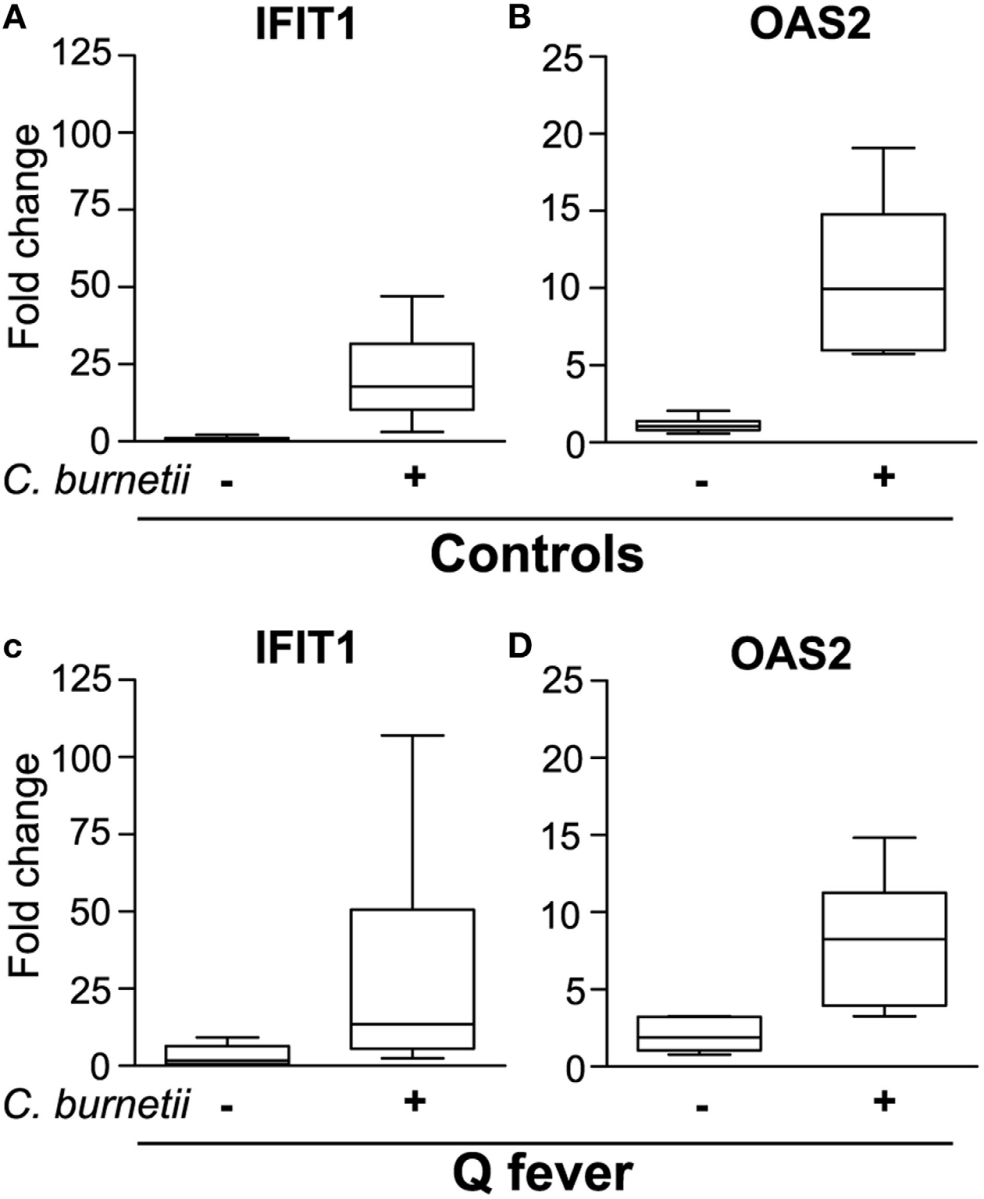
**Expression of ISGs in granulomas generated in Q fever patients**. PBMCs from healthy donors **(A,B)** and patients with Q fever **(C,D)** were stimulated with heat-killed *C. burnetii* for 9 days and analyzed for the expression of IFIT1 **(A,C)** and OAS2 **(B,D)** by qRT-PCR.

We finally inquired whether the granulomatous response of patients with Q fever was associated with alteration in type 1 IFN pathway. First, we developed a simple method to obtain granulomas by incubating control PBMCs with *C. burnetii* to simplify the recovery of isolated granulomas using *C. burnetii*-coated beads. Cell aggregates were observed after a few days. Their size progressively increased and was greatest at 8–10 days, demonstrating that the time course of granuloma formation was similar when granulomas were generated *in vitro* using beads coated with bacterial extracts (Delaby et al., 2012) or heat-killed bacteria. We investigated the expression of *IFIT1* and *OAS2*, two genes belonging to type 1 IFN pathway which were specifically modulated in *C. burnetii*-induced granulomas. The expression of *IFIT1* (Figure 4A) and *OAS2* (Figure 4B) genes was strongly upmodulated in stimulated PBMCs, but not in unstimulated PBMCs. We noted that the levels of expression of *IFIT1* and *OAS2* genes were similar to those obtained with isolated granuloma cells (see Figure 3C). The expression of *IFIT1* genes (Figure 4C) and *OAS2* genes (Figure 4D) was similar in *C. burnetii*-induced granulomas from patients with Q fever and healthy controls. These results demonstrated that type 1 IFN pathway was not altered in Q fever granulomas.

## Discussion

The favorable outcome of Q fever is associated with the presence of granulomas (Raoult et al., 2005), but the real functions of granulomas during *C. burnetii* infection remain unknown. Therefore, we employed a technique that generated *in vitro* human granulomas (Delaby et al., 2010), then performed whole genome transcriptional profile of *C. burnetii*-induced granulomas and we compared it to that induced by BCG.

More than 50% of genes that were modulated in granulomas, were commonly modulated by *C. burnetii* and BCG. First, they included genes involved in chemotaxis, especially those related to the recruitment of monocytes and lymphocytes, such as *CCL2* (Loetscher et al., 1994), *CCL8* (Loetscher et al., 1994), *CCL13* (Garcia-Zepeda et al., 1996), *CCL17* (Cronshaw et al., 2006), and *CCL18* (Adema et al., 1997) genes. This finding is consistent with the critical role of the recruitment of monocytes and lymphocytes in *in vitro*-generated granulomas (Delaby et al., 2012). Our data extend to human granulomas the role of chemokines initially described in animal models (Chensue, 2013). Hence, in mice deficient for CCR2, a receptor for CCL2 and CCL8, the number and size of granulomas, as well as monocyte recruitment at site of infection, are decreased (Jinnouchi et al., 2003). We provided evidence that the neutralization of CCL2 was sufficient to prevent the formation of granulomas in response to *C. burnetii* and BCG. As reported above in CCR2 deficient mice, it is likely that CCL2 is involved in the initial stages of granuloma formation. Similar results were obtained with the neutralization of CCL5. This result may be related to the model of CCL5 ko mice in which granuloma function is transiently impaired (Vesosky et al., 2010). In addition, granuloma cells in intestinal tissues from patients with Crohn's disease express CCL5 (Oki et al., 2005). It has been suggested that CCL5 via its interaction with CCR5 augments type 2 granuloma formation (Chensue, 2013). The role of CCL2 and CCL5 in granuloma formation in response to *C. burnetii* is likely not redundant. We hypothesize that a temporal regulation of chemokines is necessary for granuloma formation. Second, granulomas are essentially composed of macrophages, which were similarly polarized in response to *C. burnetii* and BCG. Indeed, granuloma cells expressed M1 profile with some features of M2 cells. This finding is markedly distinct from isolated macrophages infected with *C. burnetii* that express an atypical M2 profile (Benoit et al., 2008). This difference between isolated macrophages and macrophages involved in a functional unit such as granulomas has been reported in mice infected with *Mycobacterium tuberculosis*. Indeed, granuloma-associated macrophages are polarized into M1 cells whereas macrophages surrounding granulomas shift from an M1 to an M2 profile (Redente et al., 2010). In addition, the loss of M2 macrophages during *Leishmania major* infection delays disease progression and increases resistance to pathogens (Hölscher et al., 2006). We hypothesize that the microenvironment of granulomas sustains M1 polarization to eradicate pathogens.

The transcriptional response of *C. burnetii*-generated granulomas was, in part, specific. First, inflammatory genes were differentially modulated. Indeed, *C. burnetii* downmodulated the expression of *TNF*, *NCR3*, *EDN1* and genes related to the Th1 profile, such as *IFNG, TBX21*, which encodes the transcription factor tbet, *IL18RAP*, *IL26*, and *HOPX* genes. Murine macrophages infected with *Leishmania major*, a granuloma-inducing pathogen, are unable to produce EDN1 (Wahl et al., 2005). A dramatic decrease in inflammatory cytokines (IFN-γ, IL12-p40) was observed in granulomas from BCG-vaccinated guinea pigs induced by *M. tuberculosis* (Ly et al., 2007). Second, *C. burnetii* specifically induced the expression of *CH25H*, *TNFSF13*, and *IRF7* genes, known to be related to type 1 IFN pathway (Park and Scott, 2010; Tezuka et al., 2011) and likely involved in microbicidal response of granuloma cells. For instance, CH25H is an enzyme involved in production of oxysterol, 25-hydroxycholesterol. Oxysterols are known to bind liver X receptor (LXR) and its deficiency is associated with loss of microbicidal competence and apoptosis (Joseph et al., 2004). TNFSF13 likely plays a role in Th1 polarization and TNFSF13-deficient mice exhibit defective bacterial clearance (Xiao et al., 2008). Taken together, these results suggest that the activation of inflammatory genes may limit *C. burnetii* infection in specific granulomas directly or indirectly via type 1 IFN pathway.

The granuloma cells induced by *C. burnetii* exhibited upmodulated expression of numerous genes belonging to type 1 IFN pathway; most of these genes were ISGs. Although ISGs have been associated initially with antiviral response (Schoggins et al., 2011), many recent studies report their upregulation in response to bacteria and bacterial components, such as lipopolysaccharide (Textoris et al., 2012). Type 1 IFN signaling has also been implicated in efficient clearance of group B *Streptococcus*, *S. pneumoniae*, and *Escherichia coli* (Textoris et al., 2012). Indeed, injection of *C. burnetii* components in mice induces a transient IFN-α/β production, and a steady synthesis of OAS for several days (Zvilich et al., 1995). This finding is potentially important for the defense against *C. burnetii* since type 1 IFN was either not detected or its pathway was defective in macrophages or dendritic cells stimulated by *C. burnetii*, respectively (Gorvel et al., 2014). This may be related to previous reports, which state that a BCG mucoprotein is unable to induce the expression of interferon-inducible antiviral proteins (Ishii et al., 2005). The stimulation of type 1 IFN pathway (*OAS2* and *IFIT1* genes) was similar in granulomas from healthy controls or patients with Q fever. ISGs may therefore play an important role in the anti-infectious activity of *C. burnetii* granulomas.

In conclusion, the transcriptional response of *C. burnetii* granulomas includes a common part shared with other infectious granulomas and a part specific for *C. burnetii*. The specific response to *C. burnetii* involved the activation of genes involved in inflammation including type 1 IFN related genes, microbicidal activity, and apoptosis. This transcriptional program may account for the granuloma-mediated elimination of *C. burnetii*, in accordance with the natural history of Q fever where granulomas are associated with a protective immune response and resolution of the disease, whereas the absence of granulomas is associated with the chronic evolution of the disease.

### Conflict of interest statement

The Guest Associate Editor, Benjamin Coiffard, declares that, despite having collaborated with author, Aurélie Daumas, the review process was handled objectively. The authors declare that the research was conducted in the absence of any commercial or financial relationships that could be construed as a potential conflict of interest.

## Supplementary material

Figure S1 **Functional annotation of *C. burnetii*- and BCG-modulated genes**. The expression of the modulated genes in *C. burnetii*- and BCG-induced granulomas was compared to their expression in PBMCs. The functional groups that contain the largest number of distinctly modulated genes were selected, and genes were depicted. The direction of gene regulation is color-coded, with blue indicating downregulation, and red indicating upregulation.
