# Supplementary figures and images for: Granulomatous response to Coxiella burnetii, the agent of Q fever: the lessons from gene expression analysis
Source: Front Cell Infect Microbiol. 2014 Dec 15;4:172. doi: 10.3389/fcimb.2014.00172 (PMC4266094; doi:10.3389/fcimb.2014.00172)

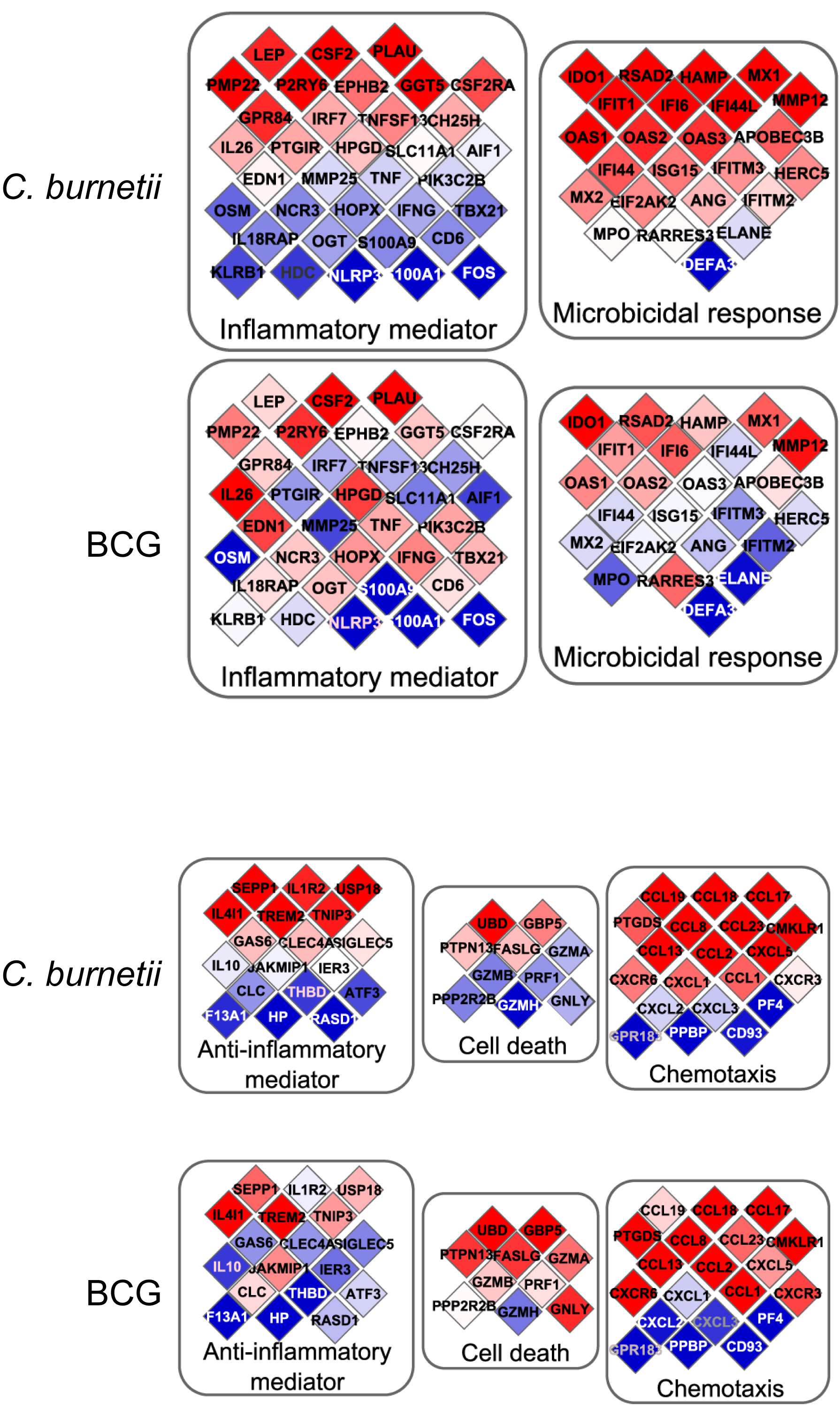

Supplement: Figure S1 — Functional annotation of C. burnetii- and BCG-modulated genes. The expression of the modulated genes in C. burnetii- and BCG-induced granulomas was compared to their expression in PBMCs. The functional groups that contain the largest number of distinctly modulated genes were selected, and genes were depicted. The direction of gene regulation is color-coded, with blue indicating downregulation, and red indicating upregulation. [file Image1.TIF]
